# Reasons for no-show to referrals at a university eye clinic after eye examination via a mobile ophthalmic unit in a Brazilian region

**DOI:** 10.5935/0004-2749.20210092

**Published:** 2025-08-21

**Authors:** Marcelo Abrão Rezende, Mitsuo Hashimoto, Carlos Roberto Padovani, Silvana Artioli Schellini

**Affiliations:** 1 Ophthalmology Department, Faculdade de Medicina de Botucatu, Universidade Estadual Paulista “Júlio de Mesquita Filho”, Botucatu, SP, Brazil; 2 Biostatistics Department, Instituto de Biociencias de Botucatu, Universidade Estadual Paulista “Júlio de Mesquita Filho, Botucatu, SP, Brazil

**Keywords:** eye health services, mobile health units, health services accessibility, Patient dropouts, Health promotion, Serviços de saúde ocular, Unidades móveis de saúde, Acesso aos serviços de saúde, Pacientes desistentes do tratamento, Promoção da saúde

## Abstract

**Purpose:**

This study aimed to identify patient’s reason for no-show at a university eye clinic after ophthalmic examination via a mobile ophthalmic unit, which provides comprehensive ophthalmic care to underserved communities in a region of Brazil.

**Methods:**

In 2017/2018, this prospective observational study searched for no-shows at referrals to a university eye clinic after an outreach program screening via a mobile ophthalmic unit in 10 municipalities in the central-western region of São Paulo, Brazil. A total of 1,928 patients underwent a comprehensive eye examination at no cost, and 37.1% of them needed referral to a university eye clinic for specialized examinations or surgeries. We used the following two main tools: (1) comparative analysis between patients who attended the referral and those who did not; (2) active search using a questionnaire to assess reasons for no-show.

**Results:**

Attendance to referrals was not influenced by age, gender, distance from the university hospital, number of ophthalmologists in the municipality, average family income, and visual acuity. The main cause for referrals was cataract (350 cases). No-show was most common among glaucoma/ suspected glaucoma (54.1%) cases, followed by strabismus (45%) and anterior segment disease (33.6%) cases. Many patients who did not attend the referral sought another service.

**Conclusion:**

Patient’s issues and lack of knowledge regarding their ophthalmic condition are the main reasons for no-show at referrals for free ophthalmic care. Thus, educational campaigns are needed to achieve consistently high attendance to prevent avoidable blindness.

## Abstract (pt)

**Objetivo:**

Avaliar as razões para não comparecimento à clínica oftalmológica da universidade após triagem oftalmológica realizada usando uma unidade móvel oftalmológica que fornece exame oftalmológico para comunidades não assistidas em uma região do Brasil.

**Métodos:**

Foi realizado um estudo observacional prospectivo no ano de 2017/2018 para avaliar as razões que fizeram com que os indivíduos triados usando uma unidade móvel oftalmológica e referenciados para a clínica oftalmológica da universidade não comparecessem à consulta agendada. A triagem foi feita em 10 municípios da região centro-oeste do estado de São Paulo, Brasil. Todos os 1.928 participantes fizeram o exame oftalmológico sem custo e 37,1% deles necessitaram de encaminhamento para a clínica oftalmológica da universidade para exames especializados ou cirurgias. O estudo usou duas ferramentas: (1) análise comparativa entre os dados dos indivíduos encaminhados que compareceram ao agendamento com os que não compareceram; (2) busca ativa dos indivíduos que não compareceram à consulta agendada, aplicando-se um questionário para avaliar os motivos para o não comparecimento.

**Resultados:**

Fatores como idade, sexo, distância entre a cidade de origem e o hospital universitário, número de oftalmologistas na cidade de procedência, renda familiar média e acuidade visual não influenciaram no comparecimento ao encaminhamento. Catarata foi a maior causa para o encaminhamento (350 casos). O não comparecimento foi maior nos portadores de glaucoma/glaucoma suspeitos (54,1%), estrabismo (45%) e afecções do segmento anterior (33,6%). Muitos indivíduos que não compareceram ao serviço de referência procuraram por outro local para o atendimento oftalmológico.

**Conclusão:**

O não comparecimento para tratamento oftalmológico sem custo depende de fatores relacionados ao paciente ou à falta de conhecimento das próprias condições oftalmológicas. Campanhas educativas nas comunidades assistidas devem ser feitas para alcançar maior comparecimento às consultas e melhor prevenir a cegueira evitável.

## INTRODUCTION

Ophthalmic care depends on trained professionals and specialized equipment, which is often unavailable at primary health care units in Brazil. Consequently, numerous ophthalmic problems are left unattended by many small municipalities, thereby increasing the demand for ophthalmic care.

Mobile ophthalmic unit (MOU) is a relative innovative care delivery model created to increase healthcare accessibility and improve health outcomes. By using MOUs, health disparities may be alleviated in vulnerable populations and individuals with eye diseases.

Fifteen years ago, in Brazil, the Medical School of State University of Sao Paulo (UNESP) started an outreach program developed by residents and attending physicians of Ophthalmology. This program uses an MOU that is equipped for comprehensive eye examinations, including refractive examination and screening for the main causes of blindness, to make eye care more accessible to underserved communities and reduce the demand for referral of simple cases to the university eye clinic.

By opening their doors directly into communities and existing community assets through the MOU, affordable eye care can be offered for the most common eye needs. Therefore, only complex cases must be referred to the ophthalmic specialized centers.

Through MOU use, only 37.1% of patients required referral to the university eye clinic^(1)^.

Brazilian municipalities in our region have facilitators that coordinate, support, and transport patients being referred to the university hospital. Unfortunately, few patients attend after referrals to ophthalmic centers. In a previous study, approximately 31.7% of the referred patients failed to attend university eye clinic appointments, and roughly 50% of patients who started treatment at the referral clinic failed to keep their follow-up^(2)^.

Considering that MOUs are a relatively new alternative to other healthcare models, the theoretical de terminants of no-show must be understood. By understanding the reasons that prevent patients from accomplishing the necessary treatment, strategies that maximize attendance can be established to prevent avoidable blindness.

The present study aimed to evaluate the no-show rate in free ophthalmic care at the university eye clinic after eye screening using our MOU outreach program in a Brazilian community.

## METHODS

The study conformed to the Declaration of Helsinki, and the Institutional Review Board of the Medical School of UNESP-Brazil approved the study protocol (ID: 2.649.545).

Conducted in 2017 and 2018, this analytical prospective and observational study sought to analyze the reasons for no-show to the referrals at the university eye clinic for specialized examinations/ophthalmic surgeries of individuals who attended eye screening during an outreach program of the university.

### Study sample

In this outreach program, screening was centrally organized, and appointments were delivered in a dedicated, equipped MOU for full comprehensive eye examination. In 2015, 1,928 patients from 10 municipalities in the central-western region of São Paulo, Brazil, were examined, and 716 (37.1%) of them were referred to the university eye clinic^(1)^. They presented with ophthalmic conditions that required medical therapy, laser treatment, or even surgery.

After collecting the names and phone numbers of the referred patients, coordinators scheduled them for follow-up and arranged their transportation with costs covered by the health system. However, 227 (31.7%) of these patients did not attend to the referral^(2)^, and this group was the focus of our study.

To verify the reason for no-show at the referrals, we employed two main tools:

1. Comparative analysis of characteristics between patients who attended the referral and those who did not. These characteristics included the demographic variables (gender, age), distance from the municipality of residence to the university eye clinic, infrastructure and human resources including the number of ophthalmologists for ophthalmic care in the home municipality^(3,4)^, average family income (official Brazilian data, Brazilian Institute of Geography and Statistics)^(5)^, general health status, presenting visual acuity (VA), best corrected VA (BCVA), and ophthalmic diagnosis leading to referral.
2. Active search for no-show and application of a semi-structured interview questionnaire, which included questions on the reason for no-show, developed by the authors. In this questionnaire, several possible reasons for missing appointments were listed, and patients were asked to account for their no-show. Reasons not covered by our list could be specified in the “other” category. The questionnaire also requested information related to family infrastructure (lack of babysitter, lack of money to travel, need to remain home taking care of others) or to the individual (fear of surgery, concomitant illnesses, seeking of another service). Patients were contacted through using the contact telephone number they provided in the first assessment performed in the MOU or in the patient identification in the Family Health Care Program or Social Service of the home municipality. We attempted to call these patients thrice at different time periods and days. Only one author (MR) conducted all the telephone interviews. If the patient was not able to answer the questions via telephone call, a member of the municipality health system asked the patient in person, using the questionnaire.

The minimum sample size of questionnaire responders was 143 (63%), which met the specifications of casual participation, with 95% confidence interval (CI), a 10% estimation error, and alpha = 5%.

### Definitions

In this study, no-show was defined as a patient who did not come to the scheduled appointment. Adopted from the World Health Organization (WHO) definitions, a presenting distance VA worse than 20/400 indicated blindness, whereas a presenting VA worse than 20/40 in the better eye indicated vision impairment (ICD 11 - 2018)^(6)^.

All data were tabulated using the Excel 2016 software (Microsoft Corp., Redmond, WA, USA) and analyzed using the SPSS 22.0 software (IBM Corp., Armonk, NY, USA). Statistically significant p values of variables are presented in the tables.

## RESULTS

The no-show rate in the university eye clinic was 31.7%. The mean age of the attended patients was 57.6 ± 19.5 years (median: 62 years [1-90 years]), and most of them were females (428/59.8%). Meanwhile, the mean age of no-show patients was 56.1 ± 16.2 years (median: 62 years [1-88 years]). Age was not significantly different between no-show and attended patients. No-show was slightly higher among females (143/ 62.9%) than males.

Table 1 shows the number of individuals assessed in the MOU within the study period, number of referrals to the university eye clinic, no-show rate according to home municipality, distance between the municipality and the university eye clinic, number of ophthalmologists in each municipality, and average family income in each municipality.

**Table 1:** Descriptive number of attended, referred, and no-show patients according to the distance between the municipality of origin and the university eye clinic, number of ophthalmologists in the municipalities, and average income (Sao Paulo, Brazil; 2018)

Municipality	Attended (%)	Referred (%)	No-show	Distance (km)	Nº ophth^**^	Mean average income^#^ (R$)
Mineiros do Tietê	348 (100)	160 (46.0)	59 (36.9)	67.1	0	694.2
Piramboia	149 (100)	20 (13.4)	9 (45.0)^*^	44.2	0	549.2
Taquarituba	172 (100)	50 (29.1)	12 (24.0)	139	1	613.8
Igaraçu do Tietê	188 (100)	65 (34.6)	23 (35.4)	50.6	0	587.4
Dois Córregos	183 (100)	54 (29.5)	20 (37.0)	76.8	2	720.3
Boracéia	192 (100)	60 (31.3)	21 (35.0)	102	0	708.0
Bariri	199 (100)	113 (56.8)	19 (16.8)	110	2	771.6
Macatuba	176 (100)	47 (26.7)	22 (46.8)^*^	65.3	0	918.6
Brotas	169 (100)	36 (21.3)	15 (41.7)^*^	98.4	0	711.0
Barra Bonita	152 (100)	111 (73.0)	27 (24.3)	53.8	4	903.2

* p<0.05;

** N^o^ Ophth - number of ophthalmologists according to the Brazilian Health System Department of Informatics and Brazilian Council of Ophthamology^(3,4) #^Official Brazilian data, with GDP per capita, average per capita family income provided by the Brazilian Institute of Geography and Statistics (IBGE)^(5)^.

The need for referral according to the home municipality ranged from 13.4% (Piramboia) to 73% (Barra Bonita).

The average of no-show was balanced among municipalities, varying from 16.8% (Bariri) to 46.8% (Macatuba); no-show from Macatuba (46.8%), Piramboia (45%), and Brotas (41.7%) was higher than that from Bariri (16.8%), Taquarituba (24%), and Barra Bonita (24.3%) (p<0.005) (Table 1).

No-show was not significantly associated with the distance of the municipality to the university eye clinic (r=-0.202; p=0.575). Taquarituba was the farthest from the university eye clinic (139 Km) and had the second lowest no-show rate (24%). The second most distant municipality from the university eye clinic (100 km) was Bariri, which actually had the lowest no-show rate (16.8%). However, Piramboia was the nearest (44.2 km) but had the second highest no-show rate (45%) (Table 1).

Moreover, the number of ophthalmologists in the municipality was not associated with the need for referral (r=0.347; p=0.278). The municipality with the highest number of ophthalmologists (4 professionals) was Barra Bonita, which had the third lowest no-show rate (24.3%), followed by Bariri (lowest no-show rate [16.8%]; 2 ophthalmologists) and Dois Córregos (sixth lowest no-show rate [37%]; 2 ophthalmologists). In several other municipalities, ophthalmologists were unavailable (Table 1).

The mean average income of the municipalities was R$717.7, ranging from R$ 549.2 (Piramboia) to R$ 918.6 (Macatuba) (Table 1).

In the better eye of no-show patients, the presenting VA was 0.49 ± 0.34 LogMAR (20/60 Snellen acuity), whereas the BCVA was 0.36 ± 0.22 LogMAR (20/50 Snellen acuity). Among these patients, 12 (5.4%) were considered blind, and 48 (21.6%) were visually impaired.

Table 2 shows the number of referred and no-show patients according to the topographic ophthalmic diagn osis. The main cause of referral was cataract (350/48.9%), followed by anterior segment (131/18.3%), eyelid and lacrimal diseases (95/13.3%), glaucoma/suspected glaucoma (61/8.5%), retina (50/6.9%), strabismus (20/2.8%), and others (9/1.2%). Considering topographic diagnosis and the number of referred patients, the highest no-show rate was glaucoma/suspected glaucoma (33/54.1%), followed by strabismus (9/45.0%), anterior segment (44/33.6%), cataract (103/29.4%), eyelid and lacrimal diseases (25/26.3%), retina (9/18%), and other abnormalities (4/44.4%) (Table 2).

**Table 2:** Reason for referral to the university eye clinic and patient no-show according to the topographic diagnosis of patients assessed by a mobile ophthalmology unit (Sao Paulo, Brazil; 2018)

	Referred	No-show	p-vahie
**Topographic diagnosis**	**n (%)**	**n (%)**	
Lens (Cataract)	350 (48.9)	103 (29.4)	p<0.001
Anterior segment	131 (18.3)	44 (33.6)	p<0.001
Eyelid and lacrimal diseases	95 (13.3)	25 (26.3)	p<0.001
Glaucoma	61 (8.5)	33 (54.1)	p<0.001
Retinal disease	50 (6.9)	9(18.0)	p<0.001
Strabismus	20 (2.8)	9 (45.0)	p<0.001
Others	9(1.2)	4 (44.4)	p=0.006

The mean attendance rate was 35.8%. According to the topographic diagnosis, the number of no-show was greater than the mean attendance for glaucoma/suspected glaucoma (54.1%) and strabismus (45.0%). The questionnaire answers were obtained from 140 (61.7%) individuals who were located by the active search. Three patients were eliminated from the sample, lowering the confidence level from 95% to 94.6% and rising the alpha value from 5% to 5.6% which, according to the evidence, did not influence the results.

Based on the questionnaire answers, the reasons for no-show were as follows: search for another service (private or public) (31, 22.1%), miscellaneous reasons (17, 12.1%), inability to travel due to other diseases (16, 11.4%), death or changing of address (15, 10.7%), failure to understand physician’s explanations (14, 10%), unperceived need for the treatment (13, 9.3%), belief that their current vision was functional for daily tasks (11, 7.9%), distant location from the municipality to the referral center (9, 6.4%), and others (Table 3).

**Table 3:** Reasons for patient no-show to the referral at the university eye clinic after a comprehensive ophthalmic examination in a mobile ophthalmic unit according to the questionnaire answers (Sao Paulo, Brazil; 2018)

Reasons for no-show	Number	(%)
Search for another service	31	22.1
Others	17	12.1
Disease/inability to travel	16	11.4
Death or change of address	15	10.7
Failure to understand the explanations about the importance of referral	14	10.0
Failure to realize the necessity to undergo treatment	13	9.3
Perception that actual vision is enough for daily activities	11	7.9
Distant location of the referral center	9	6.4
Provision of care to another ill person in the family	6	4.3
Concomitant disease that made eye surgery impossible	4	2.9
Lack of travel companion	2	1.4
Fear of surgery	2	1.4

p<0.001

## DISCUSSION

Based on two previous studies^(1,2)^, the present study verified the reasons for no-show to referrals that prevented 227 individuals in need of specialized eye care from receiving treatment at the university eye clinic. Patients who do not attend the referral center can be considered vulnerable, and reasons for no-show must be identified to promote efficacy in preventing blindness.

A potential key limitation of this study was 2 to 3 years gap since the care was delivered by the MOU (in 2015) and the questionnaire was answered (in 2017/2018), increasing the chances of recall bias and decreasing the ability to locate the research subjects. Reasons for no-show were only ascertained by 61.7%, and such reasons among patients whom we failed to contact may differ from those who were successfully contacted^(7)^.

Despite these limitations, the study outcomes provide important data for future actions. As clearly shown, factors such age, gender, distance to the university eye clinic, number of ophthalmologists in the municipality, and average income slightly influenced no-show prevention. However, several risk factors for poor attendance were linked to patient’s misunderstanding of the eye diseases and fear of the procedures or the logistic of care providers; nonetheless, such factors can be potentially modified by providing better education and a promising methodology that would enable prediction and reduction of no-show. These factors should also be considered in the prediction model.

In this study, the no-show rate was 31.7%, which was higher than 18.8% observed in a general medical clinic in the USA^(8)^ and 23.4% in an ophthalmic university program in another USA region^(9)^. However, the attendance rate found in our study (68.3%) was similar to that of other USA ophthalmic programs involving only retinal diseases, with a rate of 72% for the recommended follow-up^(10)^.

Our study is unique according to several aspects. Instead, of providing screening or triage using an MOU, physicians provide comprehensive eye examinations to patients at a hosting site, solving most common eye cases and referring to the university eye clinic for more complex ophthalmic diseases that require propedeutic exams/therapy, laser treatment, or surgery. After the MOU screening, social service coordinators from the municipalities schedule referred patients for appointments. Patient transportation, follow-up at the university ophthalmic clinic, and all stages of care are provided with no costs for the patients. Despite all these conveniences, no-show has been still observed; therefore, we decided to evaluate the reasons for such.

In many healthcare systems, no-show is a prevalent problem. Possible reasons for no-show can be inferred by comparing patients who attended with those who did not.

Factors such as age, sex, distance between the municipality served by the MOU and the reference center, number of ophthalmologists in the municipalities, average family income, and VA did not directly influence attendance at referrals. In other studies, no-show of general and vulnerable populations is mainly caused by transportation/geographic barriers, financial costs, insurance status, legal status, linguistic and cultural barriers, lack of healthcare providers, perceived absence of patient-centered care, psychological barriers, intimidation in healthcare settings, center operating hours, and anonymity concerns^(11)^.

However, many of these barriers are absent in our area, especially cost-related issues, which can be excluded from our survey because all costs are covered by the health system, even transportation.

The mean age of patients who attended (57.6 ± 19.5 years) was similar to the age of no-show patients (56.1 ± 16.2 years) (p=0.332) in referrals to the university eye clinic, revealing that age was not a relevant reason for no-show. However, taking into consideration only the elderly, age can be a potential factor causing higher no-show^(8)^.

Most of the no-show individuals were female. However, female predominance was observed in the original study^(1)^, indicating a selection bias.

The municipalities involved were significantly varied in terms of no-show at referrals, ranging from 46.8% (Macatuba) to 16.8% (Bariri). The distance of the municipality was not directly related to the no-show rate. Despite being nonsignificant, a negative association was observed between farther distances of no-show’s homes from the referral center.

In aneother study, treatment uptake was negatively associated with driving time to the nearest clinic, mainly among those patients aged ≥60 years^(12)^. However, our patients did not need to drive to receive ophthalmic care because they usually rode a bus going to the university hospital.

Furthermore, we evaluated the number of ophthalmologists who lived in the municipalities; however, this number may not be factual because some municipalities received locum ophthalmologists. According to the results, ophthalmologist availability in the municipality seemingly had no influence on the no-show rates. The two parameters, namely, availability and accessibility, are not necessarily correlated, considering that private physicians may be available in the municipality and the need for expending money can reduce accessibility.

To determine if financial problems would interfere in the no-show rates, we evaluated the average income of the municipality. However, individual socioeconomic status was strongly associated with eye care uptake in which upfront costs are high. However, evidence for this association is equivocal, given that costs are lower^(12)^ or at no costs, similar to our program.

Regarding the presence of blindness or visual impairment, no difference was found between those who attended and those who did not, revealing that this factor was not a determinant of no-show.

In relation to the number of referred individuals from each topographic ophthalmic diagnosis, patients with glaucoma/suspected glaucoma had the highest no-show rate (54.1%). Possibly, patients did not understand the disease and its treatment; thus, they were less likely to attend follow-ups. However, even patients who understand the risk of blindness may become no-shows because of the following reasons: they disregard the seriousness of their disease, they are asymptomatic (except acute glaucoma), central vision is conserved until the last phase of the disease, and they received no clear information about appointments for follow-up; only those who understand glaucoma severity are more likely to attend^(13)^. Notably, glaucoma no-shows are considerably numerous, revealing the urgent need for health education programs to mitigate the incidence of visual impairment and blindness secondary to glaucoma in the region.

The no-show rate for strabismus was also extremely high (45%). Although strabismus is caused by amblyopia, it often affects children, making people who are less aware of the disease wait longer to seek medical help.

Regarding cataract, most referrals were related to cataract surgery need. However, 29.4% of referred patients did not attend the referral despite being aware that the surgery could result in visual recovery, as evidenced by the high cataract prevalence worldwide^(7)^. No-show was mostly related to fear of surgery.

Moreover, the no-show rate for retinal diseases was 18%. The most important diseases in this group were diabetic retinopathy and age-related macular degeneration. Considering that the retinal treatment is generally expensive and only a few places provide free-of-charge procedures, adherence to referrals to a university eye clinic at no cost can be higher. However, many patients with diabetes are asymptomatic, have good diabetes control, vaguely understand diabetes complications, have wrong perceptions on retinal procedures, have conflicting appointment timetables for diabetes, and were not aware of the importance of follow-up^(7,14)^. Hence, a clear and shared understanding of the importance and procedures for screening diabetes complications would facilitate their attendance.

Eye care uptake among people with age-related macular degeneration and in the general population is positively associated with the number of ophthalmologists per capita of population, a measure of service availability^(12)^.

The present study was mainly challenged by contacting patients to respond to the questionnaire. After up to three telephone calls, we were able to include 140 individuals in different time periods and days. Similar to a previous study^(1)^, many of the individuals’ telephone numbers were inaccurate. In addition, neither the family health care or the health secretariats of the respective municipalities were able to locate no-show patients. These factors may represent a recall bias.

In this study, seeking another service (22.1%) was the main reason for no-show, suggesting a positive indication that consultation through the MOU stimulated patients to actively seek treatment. As another factor, patients going to the MOU intended to have their complaints resolved in the unit itself (68.3%). Perhaps, patients did not expect diagnoses requiring surgery, and upon receiving their diagnoses and referrals, they may have sought care closer to home. Lastly, patients may have gone to an MOU merely seeking a second opinion.

The significantly high number of no-shows in the current study may reflect the limitations of the family structure, patient’s physical conditions, or lack of information on their health condition. In one study, missed appointments are caused by the following reasons: lack of transportation (34.8%), work conflicts (34.8%), illnesses (26.1%), dependent care (13%), and other causes (21.6%)^(9)^. Enablers of attendance could operate at multiple levels in the healthcare system, such as individual, group or team, an overall organization, and a wider system or environment^(15)^. Reasons for no-show may be partly related to communication flaw, work commitments, appointment cancellation, transportation, or administrative problems. Moreover, patients may not have received the appointment date or have forgotten it, may misunderstand that further attendance was necessary, and may perceive that their experience as an outpatient is upsetting with no wish to have follow-up visits^(16)^. Other reasons for no-show could be an unwell status, retirement status, clerical error, difficulty in finding transportation, and other preventable causes, such as poor communication^(8,17)^.

An attendance-influenced subjective factor, which is generally difficult to measure, is related to the quality of service provided by the MOU. It is connected with doctor-patient dynamics established during MOU visit. A poor doctor-patient relationship can influence patient attendance at the referral.

The current study is beneficial because it detected reasons for no-show at a free ophthalmic care program in our region, providing directions for future actions that can improve blindness prevention.

In conclusion, no-show at an ophthalmic referral center after a comprehensive ophthalmic evaluation in a MOU depends on factors related to the patients (general health, family structure) or lack of knowledge of ophthalmic conditions. Hence, education and awareness campaigns in MOU-assisted communities should be implemented to improve attendance at the proposed treatments to prevent avoidable blindness.
